# Challenges of Confidentiality in Clinical Settings: Compilation of an Ethical Guideline

**Published:** 2018-06

**Authors:** Mahshad NOROOZI, Ladannaz ZAHEDI, Fataneh Sadat BATHAEI, Pooneh SALARI

**Affiliations:** 1. Medical Ethics and History of Medicine Research Center, Tehran University of Medical Sciences, Tehran, Iran; 2. Dept. of Medical Ethics, School of Medicine, Tehran University of Medical Sciences, Tehran, Iran

**Keywords:** Confidentiality, Clinical setting, Confidentiality guideline, Medical ethics

## Abstract

**Background:**

Respecting patients confidentiality and privacy are considered as the patients’ rights. Confidentiality is the key virtue for trust building in physician-patient relationship. While law considers confidentiality as absolute except for legal situations, despite efforts to maintaining confidentiality, sometimes breaching confidentiality is unavoidable but not necessarily unethical. There is no Iranian unified ethical guideline to define clear approaches to patient confidentiality in clinical setting. To keep all medical data confidential it is necessary to identify the scope of the problem. In this study, we aimed at identifying the scope of the problem.

**Methods:**

This study was conducted in three phases including literature review, qualitative study (semi-structured interview) and focus group discussion. The literature review provided a framework for the second phase.

**Results:**

The content analysis of the interviews presented 3 main themes indicating problems in maintaining confidentiality in clinical setting including management issues, organizational ethics and physician-patient relationship.

**Conclusion:**

Based on the results a draft guideline in confidentiality in clinical setting was prepared and finalized in focus groups discussions.

## Introduction

Since Hippocrates, confidentiality has been presented as 1 cornerstone of ethics in healthcare. Confidentiality roots back to the respect for autonomy and self-control on information. Respecting patients confidentiality and privacy are considered as the patients’ rights. From deontological aspect, confidentiality is a duty and based on virtue ethics which Islam insists on; maintaining data privacy and confidentiality is the key virtue for trust building in physician-patient relationship. In healthcare settings patient’s information should be kept confidential in professional relationship (1).

The patients’ medical information is not only what the physician obtains during objective observations, clinical examinations, and test results but also his/her perceptions about family life, lifestyle, and habits as well. Inappropriate disclosure of that information may threat patient’s reputation, opportunities, and human dignity. Physician-patient relationship is generated based on the trust between the two parties. A patient may share confidential information (stressful, embarrassing, and harmful) with physician needed to get an accurate diagnosis. This means that there should be a mutual trust between the two; especially when encountered a mentally disordered case (2). The patient’s concern about keeping confidentiality has drastic impact on their trust (3). The patients may conceal some information from physician, and less likely to refer to the physician for treatment or follow up (4) especially who gets familiar with privacy concerns of them through new technologies including mobile apps and internet sharing.

Some consider respect for confidentiality as an absolute duty for physicians, but in reality, the absolute maintenance of confidentiality is not always possible and there exist several exceptions (5). In general, ethical guidelines define certain circumstances at which relative or absolute maintenance of confidentiality is possible.

The Iran law strongly protects confidentiality; as Iran’s Islamic Penal Code (IPC) (article 648) sentences penalties for breaching confidentiality except for legal justifications. Moreover, the Disciplinary Regulation of Iran’s Medical Council prohibits breaching confidentiality. Despite the laws and regulations, no Iranian unified ethical guideline has defined a clear framework for patient confidentiality in clinical settings.

To keep all medical data confidential, it is necessary to identify the scope of the problem. In this study we aimed at identifying the scope of the problem including the concerns declared by physicians about confidentiality in clinical settings, then through problem-solving; we proposed an ethical guideline for physicians in this regard.

## Methods

### Study design

The study was conducted in Medical Ethics and History of Medicine Research Center of Tehran University of Medical Sciences, Tehran, Iran from November 2014 until March 2015. This qualitative study was performed in three phases: Phase one: A gap analysis study was performed and some of the most relevant guidelines and papers were reviewed. Our search was limited to the most recent papers and guidelines in English and Persian. Moreover, some of the national guidelines from United States, UK, Canada, etc. were included in the study. We gained a complete understanding of the meaning and implications of confidentiality and recognized different dimensions of the issue and the challenges around it. In addition, we identified some social, cultural, and religious aspects based on the views of the physicians/interviewees. Finally, the gap between theory and practice and the ethical challenges of confidentiality was characterized and an interview guide for the second phase of the study was generated.

Phase two: Semi-structured face to face interviews to specify the views of the clinicians about the challenges of confidentiality in clinical setting, were performed. The interviewees’ selection was purposive; the study participants were selected among clinicians who were specialist in different disciplines.

At the beginning of the interview each study participant was informed about the study and its aim, ensured about their confidentiality and voluntariness and oral consent was obtained from participants. All interviews were audio recorded and continued until data saturation.

The interviewees opinion regarding the concept and importance of confidentiality, relative and non-relative confidentiality, confidentiality in medical consultation and data sharing, state of breaching confidentiality and the one who is responsible for keeping confidentiality in health care system, confidentiality in grand rounds and morning reports, confidentiality after death, confidentiality in social media, and electronic records, confidentiality in children and adolescents, confidentiality in professional relationship among healthcare providers especially physician-colleague relationships, confidentiality in detention settings and child abuse, and technological, and structural obstacles to acceptability of data sharing were subjected to content analysis. Congruent and incongruent perspectives were probed among the participants.

Phase three: In-depth discussion about themes of interviews through FGD, compiling the preliminary draft of the guideline was conducted. In this phase, a draft of the guideline was assessed through an in-depth focus group discussions. The ethicists and clinicians of the department of medical ethics of the Academy of Medical Sciences of Islamic Republic of Iran participated in the focus groups. Accordingly, their comments were reviewed and the changes were done and the preliminary draft of the guideline for keeping confidentiality in clinic was compiled.

### Data analysis

All the interviews were transcribed verbatim more than three times to get familiarize with data through frequent readings and note takings. The original challenges were extracted, defined and classified as the main themes and subthemes.

### Ethics

This study was reviewed and approved by the Institutional Review Board (IRB) of the Academy of Medical Sciences, Islamic Republic of Iran.

## Results

### Phase one: Gap analysis

**Fig. 1:**
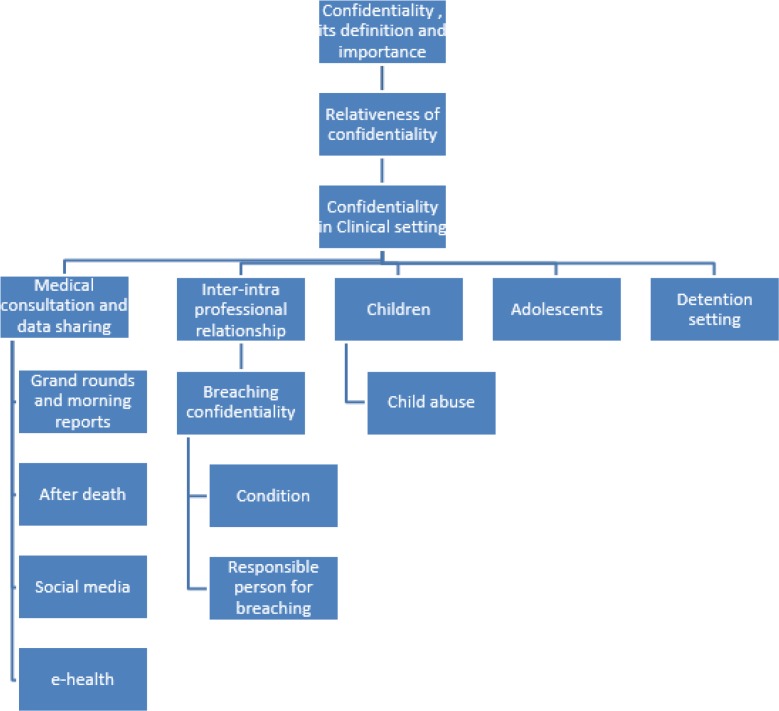
The framework for interview based on the results of literature review

The results of the literature review are summarized in Fig. 1.

### Phase two: Interviews

Using a professionally representative sample of clinical specialists including 2 gynecologists, 3 pediatricians, 2 internists and one endocrinologist, this phase was conducted. The interviewing guide comprised open-ended questions. Confidentiality was respected by omitting all interviewees’ identifiable information from results. All of the main generated themes were categorized in three levels including challenges of confidentiality in clinical settings related to management, organizational ethics and physician-patient relationship (Table 1).

**Table 1:** The challenges of confidentiality in clinical practice

	***Challenges***	***subthemes***	***Examples***
1	Management issues	Insufficient Laws and regulations Insufficient policies	Lack of national confidentiality guideline, lack of transparency in IPC and disciplinary regulations, no definition of patients benefit or the benefit of society, no clarification about physicians responsibilities, Lack of transparent policy, lack of implementation mechanisms, lack of monitoring mechanisms
2	Organizational ethics	Insufficient organizational regulations Improper infrastructure Human resources Technologic impairment	Lack of transparent regulations for management of data publication, data accessibility; non-efficient hospital ethics committees, non-awareness, data safety in cyberspace
3	Physician-patient relationship	Service providers Meanings & measurements Healthcare recipient	Gap of knowledge, insufficient attention, paternalism, physicians judgment, no definition of patients risk, and emergency vs. non-emergency situations, patients awareness,

### Challenges in Management

Insufficient law and lack of regulations have left ethical challenges of management unsolved. There is only no regulation but also no clarification in circumstances in which confidentiality is not absolute.

Some of the ethicists’ turns to patients benefit while some other consider the risk imposed to the third party, in their decision-making and solving the dilemma. [*Data sharing for the sake of patients benefit is acceptable; sometimes it is permitted without patients’ consent.*] (Participant number 2)

[*To respect confidentiality the physician should balance between the benefit of the society and the benefit of the patient.*] (Participant number 2)

[*Sometimes keeping confidentiality depends on the type of the disease for example when the patient is HPV positive there is no need to inform her husband but when she is HIV or HBS positive and the health of her husband is at stake we have to inform him; of course at first I inform my patient of the disclosure*.] (Participant number 5)

[*Sometimes saving our patients life has priority over confidentiality so we may disclose some of their information to get some help*.] (Participant number 1)

Obviously, none of the participants could define precisely the level of patients benefit nor the risk imposed to the third parties.

In clinical practice, confidentiality is not always an absolute rule, but there is no clarified policy or regulation to frame the situation in which breaching confidentiality without patients consent is acceptable, nor to determine the responsible person for disclosure.

[*The responsible person for disclosure in a right situation should be determined; the physician is responsible and all of the hospital staff should be educated about that.*] (Participant number 2)

[*Confidentiality is not taught to our residents and we as their mentors do not have enough expertise*.] (Participant number 1)

[*At residency I had a classmate who was HBS Ag+, when there is the risk of virus transfer, I disclosed his problem to others*.] (Participant number 1)

### Challenges in Organizational ethics

The other main problem in our health system is the secure process of electronic health recordings. While the patient’s information is documented as electronic documents all health care providers may have access to the records by using their own password; however, if they forget theirs, they can use the others passwords. There is no policy defining the level of password strength or no tracking system to control who has the access to patients’ information.

[*I do not know how much of information should be accessible in electronic health records but I think it is necessary to determine its limits*.] (Participant number 2)

[*To keep confidentiality, I personally do not fill in completely the patients’ documents specifying some sensitive information.*] (Participant number 2)

[*All of the patients’ data is accessible through our e-health system and most of the time the health care providers use each other’s password because they forget their own*.] (Participant number 2)

[*Because of the risk of unauthorized data sharing, I prefer not to document patients sensitive information such as addiction, HIV state, etc*.] (Participant number 5)

[*The level of data availability should be defined in e-health system for each health care provider*.] (Participant number 6)

In the recent years, the cyberspace is another threat to maintaining confidentiality.

[*Exchange of information in the cyberspace is a serious problem, although there is no regulation for that in our country*.] (Participant number 2)

[*Using cyberspace help in patients’ diagnosis and management is accepted even if patients’ information is shared*.] (Participant number 1)

[*Ethics makes a framework for inter-professional relationships and should be supported by law*] (Participant number 2)

### Challenges in Physician-patient relationship

There is no clear boundary and framework for keeping confidentiality which necessitates it’s clarifying through an appropriate national policy. Because of this necessity and also because many of the patients are not aware of their rights, most of the times the physicians do not observe the importance of maintaining confidentiality. In addition, many of the physicians and hospital staff are not aware of their duty of providing confidential services.

[*This is related to patients’ right, but they do not know that they even have a right to confidentiality.*] (Participant number 1)

[*This is related to a sociological matter. The physician should know the social concepts in which he/she works and think about the issue. Does disclosing patients’ data impose any more problems to the patient or it may resolve his/her problem? So the physician should decide; he should be educated about confidentiality.*] (Participant number 2)

[*Presenting patients’ information in grand rounds and morning reports is in the course of students’ benefit and the benefit of the society, so it is acceptable*.] (Participant number 2)

[*I prefer to get patients’ consent about their presentation in the morning report but my colleagues do not consider their consent*.] (Participant number 1)

Children and adolescents are two more sensitive age groups. In this part of the study, we considered the challenges of keeping confidentiality in children and adolescents in managing their health issues and physician-patient relationship. Obviously, in child abuse cases, confidential reporting to higher level authorities needs to be based on the national policies.

[*In adolescents, we should first talk to their parents and informs them about their child’s health problem. I think the law compels us to do so*.] (Participant number 1)

[*About a health problem in a 16–17 year old girl while there is a cultural concern such as virginity, I think first I have to inform her father because it may need prosecution*.] (Participant number 5)

[*Nowadays there are different social problems in clinics. I think if the ethics committee provides the guideline or protocol of keeping confidentiality, it would be of great help*.] (Participant number 5)

[*Informing a society about cases of child abuse depends on the social psychology although I am not agreed with it*.] (Participant number 6)

[*We do not have a guide for child abuse reporting in our country*.] (Participant number 6)

### Phase three: in-depth discussion about themes of interviews through FGD, compiling the preliminary draft of the guideline

In the last phase of this study, all categorized challenges guided us to prepare the draft of the ethical guideline for keeping confidentiality in clinical settings. This guideline is consisted of 10 articles stating definitions, and conceptualization of confidentiality, the circumstances in which breaching confidentiality is acceptable, the consequences of breaching confidentiality, the circumstances in which information disclosure is exclusively acceptable by patients consent, information disclosure at the sake of the benefit of the society, breaching confidentiality due to legal constraints, confidentiality in children and mentally debilitated patients, confidentiality in cyberspace and e-health, and confidentiality in morning reports and grand rounds.

## Discussion

Generally, respect to confidentiality has not been maintained along with developments in technology. In spite of efforts into maintaining confidentiality, sometimes breaching confidentiality is unavoidable but not necessarily unethical. The frequency index of one breach per 62.5 h was reported, most of them were severe and related to consultation with medical personnel not involved in the patients care and mostly occurred in public area (6). A significant number of breaches occur by health professionals who are aware of confidentiality but do not know the way of avoiding breaches (7).

We provide a preliminary draft for ethical guideline for maintaining confidentiality and circumstances in which breaching confidentiality is ethically accepted in clinical settings. Within this draft, three aforementioned aspects of respecting confidentiality in healthcare setting including management issues, organizational ethics, and physician-patient relationship are inserted.

The obligation of respecting confidentiality in medicine goes back to the Hippocratic Oath in 4^th^ century BC (8). Accordingly, all health care providers ought to protect patients’ information whether saved as paper print or electronic health records. Since 1996 United States of America has enacted the Health Insurance Portability and Accountability Act (HIPAA), all healthcare providers should acknowledge HIPAA for protecting patients’ information (9). The HIPAA also contains the regulations for storage, inspection of records and special strategies for prevention of data abuse (9).

In general, whenever there are some concerns about safety of the third party or public health, the absoluteness of confidentiality is questionable (10). In modern medicine data sharing, should be done after patients’ consent unless otherwise specified in related normative guidelines. Within this draft, some exceptions to absolute confidentiality without patients’ consent have been mentioned. Violations to keep confidentiality are permitted: 1) at a legally authorized request; 2) when the patient’s best interest requires it; 3) while maintaining the welfare of the society and 4) when it is necessary to safeguard the third party from a major harm or threat. In agreement with this draft, the Australian law balances the individual benefit versus safety of the society (11). The Irish Medical Council also states the conditions under which exceptions to absolute confidentiality is accepted (12).

Breaching confidentiality based on the third parity’s benefit is a major ethical challenge in respecting patients’ confidentiality and it is managed differently in different countries; however, in our country, there is no clear guideline in this regard. Both United States and the UK have similar policy on this issue. The Code of Medical Ethics of American Medical Association indicates: “The obligation to safeguard patient confidences is subject to certain exceptions ethically and legally justified because of overriding social considerations. Where a patient threatens to inflict serious bodily harm to another person or to him or herself. There is a reasonable probability that the patient may carry out the threat, the physician should take reasonable precautions for protection of the intended victim, including notification of law enforcement authorities” (13). Moreover, the General Medical Council and the British Medical Association has the same ethical considerations for British physicians (14,15). In contrast, the Code of Medical Ethics for French physicians is similar to Iran and states “Professional confidentiality instituted in patients’ interest, is obligatory for every physician within the conditions established by law”. Confidentiality applies to everything the physician earns in the exercise of his profession; that is to say not only what has been confined to him, but also what he has seen, heard or understood. “(16). Breaching confidentiality acceptable was found in psychiatric therapy from French lay people depending on several factors including consultation with an expert, and the gravity of the threat (17). Obviously, the acceptability of breaching confidentiality goes back to the magnitude of the threat to the third party. Therefore, the magnitude of the threat should be defined completely to prevent ambiguity and moral complexity. Magnitude of the threat depends on the risk severity. According to the interviewees when the risk is too severe to threaten the third parties’ lives (as Tarasoff rule) (18) breaching confidentiality is considered to be accepted.

Iran legal system mandates physicians and other hospital staffs to report child abuse and every other type of abuse as well as contagious diseases. Sometimes this legal duty is in conflict with patients’ confidentiality, therefore a proper guideline or ethical framework would be of great help. Child abuse or sexual violence causes physical and mental distress in short and long-term. The victims will suffer stigmatization, discrimination and get more sensitive to violation and sometimes become a subject of more violations. In this situation, the victim regardless of age may seek real protection of health care system especially the physicians. Reporting any case of abuse and disregarding confidentiality in report may diminish the trust and sensitizes the victim to more violations. Accordingly, the child abuse report should be based on an appropriate guideline while the patient or his/her surrogates’ consent should not be ignored. In the draft, we differentiate between different types of consents drawn from patients according to the severity of their diseases, for example, consents obtained from incapacitated patients. There is a need, however, to make further investigation into compilation of an ethical guideline for reporting child abuse adapted to our local condition, cultural and religious principles.

Adolescents have the right to confidentiality the same as adults. Privacy has an impact on adolescents-physicians relationship and lack of confidentiality could be their major obstacle to seek for healthcare (19). The Constitution of Iran considers children of 18 and above as able to give consent without their parents’ stewardship. Accordingly, their information should be kept confidential in clinical settings. For children of less than 18 there would be a challenge as we do not know their degree of maturity consistent with their autonomy and ability to decision making, so keeping their information confidential, putting them responsible, and not informing their parents about their health-related issues would be problematic. The child who understands the diagnosis was considered and treatment and makes decision as competent enough who has the right to confidentiality (20). From cultural point of view, Iranian children are under full parental support by their parents until marriage and it is likely the parents be present at all occasions in clinical settings with their child. Therefore, an ethical guideline clarifies the solution for this issue based on children’s age, their parental support, etc. to protect child’s confidentiality.

Adolescents’ high-risk behavior is the major cause of their morbidity and mortality (19). The adolescents who seek health care and report their engagement in high-risk behaviors so they might have concerns about confidentiality (21). The fear of breaching confidentiality is the major reason for not seeking health care in adolescents (22, 23) especially the high-risk ones (21). Therefore, provision of confidential health care services is necessary for them. While parents confirm the benefit of confidentiality for adolescents, they concern about assisting continuation of their child’s risky behavior by confidentiality (24, 25). The parents may be distracted about adolescents’ confidentiality and its importance (26). They would provide confidential contraceptive services to adolescents without informing their parents (27). Confidentiality concern can be a reason for waiver of health care and increase the restrictions of confidentiality may decrease health care use in adolescents which has harmful health consequences (21). Considering legal context, application of well-set procedures and requisite relationship with adolescents was necessary for respecting confidentiality and will mitigate obstacles for adolescents to refer to health care system (28).

Keeping patients’ information in electronic health records is one of the other sources of breaching confidentiality because a generic username and password are used to log in to all electronic records of all patients. Sharing username and password and also patients’ information are common among medical students, residents and all other professional academic levels. By logging in each person to the system all of the patients’ data can be easily accessed. Therefore, modification of administration based on IT security and defining different levels of access for different users is highly recommended. Because improving computer knowledge has positive effect on complying with IT security, some suggest continued education about confidentiality and continuous inspection for better confidentiality implication (29). Therefore, using smart cards, and saving files as password protected are recommended (29).

Some of the challenges we discovered are multidimensional and need more investigations from ethical, legal and social aspects. Of that confidentiality in relationship with social media, confidentiality in child abuse, designation of system audit, confidentiality in detention setting, and confidentiality after death have not been included in our draft. In fact, this draft is the first ethical guideline to confidentiality in clinical settings to address the most common ethical challenges and the scope of confidentiality; however, compilation of the later drafts for confidentiality in relationship with social media and confidentiality in child abuse is underway. Of course, some of the other actions are required to set the goal of changes in national laws which are beyond the scope of this study.

## Conclusion

Respect to confidentiality goes back to respect for autonomy and human dignity. According to Iranian law breaching confidentiality is forbidden while in some occasions in medical practice it is unavoidable. Therefore and based on our results we compiled a guideline to shape ethical decision making when facing with ethical challenges in practice. So the compiled guideline helps medical professionals to have an ethical approach toward confidentiality. However the multidimensional nature of the challenges of confidentiality in medical practice necessitates further investigations from ethical, legal and social aspects.

## Ethical considerations

Ethical issues (Including plagiarism, informed consent, misconduct, data fabrication and/or falsification, double publication and/or submission, redundancy, etc.) have been completely observed by the authors.
